# Characteristics of the tissue section that influence the staining outcome in immunohistochemistry

**DOI:** 10.1007/s00418-018-1742-1

**Published:** 2018-10-24

**Authors:** Sylwia Libard, Dijana Cerjan, Irina Alafuzoff

**Affiliations:** 1 Rudbeck Laboratory, Department of Immunology, Genetics and Pathology, Uppsala University/Uppsala University Hospital, Dag Hammarskjölds väg 20, 751 85 Uppsala, Sweden; 2 Department of Pathology, Uppsala University Hospital, Uppsala, Sweden

**Keywords:** Immunohistochemistry, Thickness of a section, Extent of staining, Pitfalls

## Abstract

Immunohistochemistry (IHC) is influenced by several factors such as cold ischemia time, fixative, fixation time, paraffin, storage time, antibody, antigen retrieval technique and detection systems. In the setting of post-mortem tissue, not only post-mortem delay, but also agonal state is of interest. Here, we assessed an additional variable, i.e., the thickness of the section, and noted that this variable also influenced the IHC outcome. This is of significance when the extent of labelling is a parameter to be assessed, for example when assigning a stage or grade of a disease. Furthermore, when assessing brain tissue with neurons, soma measuring from 4 to 100 µm, various cellular compartments composed of different proteins are localised in sections measuring 4 or 7 µm. Thus, what is seen in a 7-µm-thick section might be lacking in a 4-µm-thick section. Lack of information regarding the molecular size of commercial antibodies is also disturbing as this parameter might influence the distribution of the molecule in the three-dimensional section. The choice of antibody to be used and the staining methodology have been acknowledged being of significance for IHC outcome; however, neither sections thickness or the molecular weight has been discussed sufficiently. IHC has been shown to be an unpredictable technique used for assessment of tissue. This emphasises the need for detailed methodological descriptions in publications, the need to acknowledge and to harmonize all eventual pitfalls related to this methodology.

## Introduction

Immunohistochemistry (IHC) applied in pathology is important both in clinical practice and research (Vyberg and Nielsen 2016; Uhlen et al. 2017). With antibodies (Ab), epitopes/proteins are visualised with various IHC detection methods. Thus, a protein of interest is visualised in the tissues and in a specific cellular compartment (Ramos-Vara and Miller 2014).

The properties of Abs and their ability to bind to cells were discussed already in the end of 1800 (Ehrlich 1906). The technique was revolutionised in 1970s and 1980s by the development of enzyme-mediated IHC methods and detection systems (DS) for light microscopy (Sternberger and Sternberger 1986). Thus the profession has used this technique in research as well in clinical practice already for close to 40 years.

It is acknowledged that the IHC method is significantly influenced by the characteristics of the tissue. Factors such as cold ischemia time in surgical setting, post-mortem delay (PMD), fixation time (FT), paraffin, storage time in paraffin, storage temperature, age of the cut sections, antigen retrieval (AR) technique and DS have reported to influence the outcome of IHC (Pikkarainen et al. 2010; Skaland et al. 2010; Karlsson and Karlsson 2011; Engel and Moore 2011; Ramos-Vara and Miller 2014; Grillo et al. 2015, 2017; Lundström et al. 2018). Furthermore, in the setting of post-mortem (PM) tissue, not only PMD but also agonal state influences the IHC (Monoranu et al. 2009; Lundström et al. 2018). Many of the potential pitfalls stated above are poorly acknowledged and seldom listed in publications. Thus when reproducing a study it is almost impossible to decipher the cause when eventually producing altering outcome.

The IHC stain can be carried out on section of various thicknesses. In surgical setting, the section thickness (ST) is around 3–4 µm, whereas the ST ranges from 7 to 15 µm when assessing PM brain tissue (Alafuzoff et al. 2009; Vyberg and Nielsen 2016). In a recent study, the influence of ST in the setting of surgical samples was studied and the authors concluded that this factor is indeed of significance for IHC outcome (McCampbell et al. 2017). Noteworthy, already in 2009 Leong in an editorial pointed out that the ST should be taken into consideration (Leong 2009). Here, we assess the influence of ST on the outcome of IHC, while assessing PM brain tissue; furthermore, we address other variables that are of interest while applying IHC technique.

## Materials and methods

The regional Ethics Committee of Uppsala, Sweden has approved this study (#2013/176, updated 2016 and #2011/286, updated 2015), and written consent for scientific use of the diagnostic tissue has been given by a close relative.

**Fig. 1:**
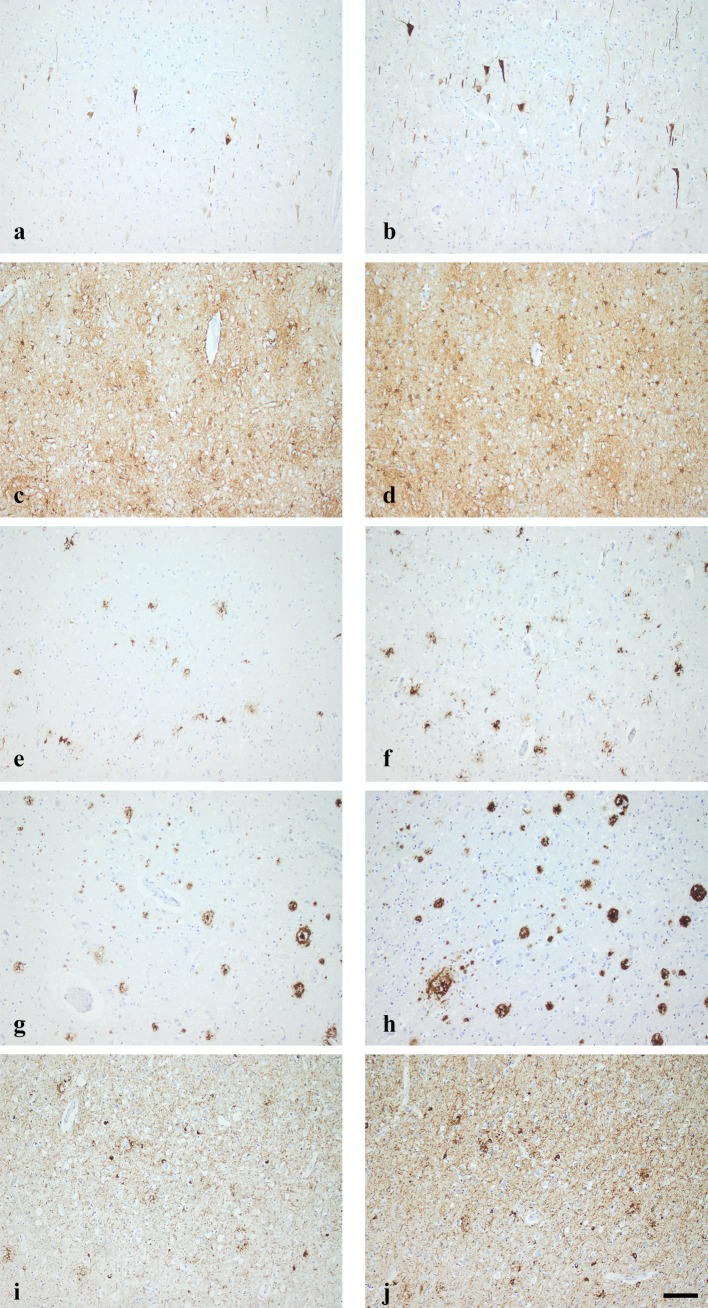
Photomicrographs of representative stains when using 4 µm (**a, c, e, g, i**) and 7 µm (**b, d, f, h, j**) thick sections. The antibodies and staining protocols as summarized in Table 1. Note the increase in labelling when comparing the left column with the right. There is seen both an increase in the number of labelled cell and an increase in labelled cell processes when applying antibodies directed to different cellular compartments as seen in **a**/**b**—SMI32, **c**/**d**—GFAP, **e**/**f**—HLA-DR and **i**/**j**—HPτ. Likewise an increase in the extent of the extracellular protein aggregates is observed in **g**/**h**—Aβ. The bar is given in **j** 100 µm

The study was carried out on PM brain tissue from a 69-year-old demented female patient with definite diagnosis of Alzheimer´s disease (AD). The brain tissue displayed characteristic features of AD including cortical extracellular β-amyloid (Aβ) aggregates and neuronal accumulation of hyperphosphorylated tau (HPτ) (Fig. 1). The PMD was 4 days and FT in 10% neutral buffered formalin (4% formaldehyde) was 46 days. The tissue samples were from the right frontal cortex, sampled according to a standardised protocol as previously described (Libard et al. 2018) and processed into paraffin (Histowax from Histolab) blocks. The blocks were stored in room temperature for 6 years. Subsequently, the blocks were sectioned into 4- and 7-µm-thick sections that were placed on Super Frost slides for standard Hematoxylin–Eosin and IHC stainings. For production on the sections of given thickness the microtome from Thermo Scientific (Microm HM 355S) with appropriate settings, i.e., four respective 7 µm was used. Before sectioning the Cool plate from Cellab Nordica was used for cooling the blocks.

**Table 1:** Immunohistochemical stains

Antibody	Clone	Company/Code	Dilution	Pretreatment
Amyloid β (Aβ)	6F/3D	Dako-Agilent/M0872	1:50	98–100% FA-2 min
CD 68	KP1	Dako/IR609		pH high
Glial fibrillary acidic protein (GFAP)	Polyclonal	Dako/ Z0334		pH high
Human leucocytic antigen -DR, α-chain (HLA-DR)	TAL.1B5	Dako/M0746	1:30	pH low
Ionized calcium-binding adaptor molecule1	Polyclonal	Wako/NordicBiolabs 019-19741	1:5000	pH high
Neurofilament H (SMI32)	SMI32	BioLegend (Sternberger) SMI32	1:1000	pH high
Synaptophysin	SP11	Abcam/ab16659	1:40	pH high
Hyperphosphorylated (Ser202/Thr205) τ (HPτ)	PHF-TAU-AT8	Fisher Sientific-Invitrogen/MN1020	1:1000	None

Dako Autostainer Plus (Dako Cytomation) was used for amyloid-β, Tau 8, SMI32, HLA-DR and Iba1 whereas CD68, GFAP and synaptophysin were stained with Dako OMNIS. Formic acid (FA)

All IHC stainings were carried out automatically according to the manufacturer’s instructions, using the Dako EnVision Flex detection system (DakoCytomation, Glostrup, Denmark). The Abs used in the study, the AR regiments applied and the IHC platforms are given in Table 1. All listed commercial antibodies have been validated by the manufacturer as given in the specification sheets.

All slides were assessed using light microscopy (Olympus BX45) at magnification 20× to 400× and then scanned into digital slides. The scanning of slides was carried out in a slide-scanning system Aperio AT2 (Leica Biosystems) in 20× magnification. The scanned figures were obtained and saved in ScanScope Virtual Slide (svs) format. Aperio ImageScope software (Leica Biosystems) was applied for the morphometric analysis, and the positive pixel count algorithm (version 9.1) was used for quantification of the immunoreactivity (IR). The Intensity Threshold (upper limit) of weak positive pixels was set to 255. Other parameters were pre-set in the software. Staining intensity was visualized by colour code where the weak staining intensity was visualized in yellow, moderate intensity in orange, strong staining intensity in brown and negative pixels in blue.

The IR was assessed within two grey matter areas measuring 4 mm^2^ each. The sum of all, i.e. weak, moderate and strong, IR positive pixels in an area was calculated and transformed into a stained area given as mm^2^. The ratio between the labelled (IR) area per total area analysed × 100 is given as stained area fraction (SAF) in percent. The reported result is the mean SAF value obtained from two assessments.

## Results

Performance of assessed Abs, as SAF, within the frontal cortex in 4 µm and 7 µm thick sections is summarised in Table 2 and visualised in Fig. 1.

**Table 2:** Stained area fraction (SAF) in percent assessed in the frontal cortex in 4 µm and 7 µm thick sections

Thickness of the section	4 µm	7 µm
Protein	SAF	
Synaptophysin	86	91
Neurofilament H Sternberger-Meyer Immunocytochemicals 32 (SMI32)	5	10
Glial fibrillary acidic protein (GFAP)	78	86
CD68	2	4
Human leucocytic antigen -DR (HLA-DR)	6	9
Ionized calcium-binding adaptor molecule1	3	5
Amyloid β (Aβ)	16	27
Hyperphosphorylated τ protein (HPτ)	61	72

The IR of the neuronal marker, synaptophysin, a glycoprotein located in the presynaptic vesicles in the nerve endings increased with ST from 86 to 91%. The IR of neurofilament H (SMI32), a neuronal cytoskeletal marker increased from 5 to 10%. The SAF of glial fibrillary acidic protein, an intermediate filament, was 9% higher in the thicker 7 µm section. Even the expression of the three microglial markers increased with ST. The CD68, a transmembrane glycoprotein located in the cytoplasmic granules increased from 2 to 4, the Human leucocytic antigen-DR (HLA-DR), a MHC class II cell surface receptor, increased from 6 to 9% and the allograph inflammatory factor localized in the cytoplasm of microglia, ionised calcium-binding adaptor molecule 1(IBA-1) increased from 3 to 5%. The SAF of Aβ increased with 41% and of HPτ protein with 15% in the 7 µm thick sections.

Thus, the IR outcome of all Abs tested here increased parallel with the increase of the ST.

Even the intensity of the staining varied in some samples, the intensity seemed to be stronger in the thicker sections. The intensity of the staining has not affected the result of the SAF as all positive pixels, weak and strong, were included.

## Discussion

We observed that the ST influenced the outcome of IHC staining in line with a previous report by McCampbell et al. (2017). This observation is of interest as the ST used in pathology varies generally from 3 to 15 µm (Alafuzoff et al. 2009; Vyberg and Nielsen 2016; McCampbell et al. 2017). Thus, in interlaboratory setting and while carrying out comparative studies including both surgical and PM samples, the ST has to be harmonised (Alafuzoff et al. 2008; McCampbell et al. 2017; Libard et al. 2018).

Based on our results, the extent of HPτ given as SAF increase from 61% seen in 4 µm thick section, to 72% seen in 7 µm thick section. This difference might have significant influence while assessing the stage of HPτ pathology in Primary Age Related Tauopathy and AD while following the current recommendations (Braak et al. 2006; Montine et al. 2012; Crary et al. 2014). All the consensus recommendations are based on ST of 7 µm, but this information is not always given (Thal et al. 2002; Braak et al. 2006). The result obtained while assessing the 4-µm-thick section might lead to lower progression stage of pathology thus interfering with the definite diagnosis. The extent of Aβ almost doubled and thus even this would interfere with the definite diagnosis while applying the current recommended staging strategy (Thal et al. 2002; Alafuzoff et al. 2009; Montine et al. 2012). In line with the above, the increase in the number of cells when applying SMI32 with the section thickness can lead to severe miss interpretations related to cell count, i.e., eventual cell loss might be unrecognized (Libard et al. 2018).

We also noted in addition to the increase of SAF an increase in the staining intensity. This is probably because more protein has been labelled in a thicker section, i.e., in a thicker three-dimensional structure. Furthermore, as pointed out by Leong, in a 4-µm-thick section when compared with a 7-µm-thick section different cellular compartment are represented, i.e., membrane, cytoplasm, or nucleus. Particularly when assessing large structures such as neurons with a soma measuring 100 µm only parts of the neuronal structures are localized in the section and thus this might significantly interfere with visualization of an epitope (Leong 2009).

BrainNet Europe (BNE) carried out several trials, with emphasis on the reproducibility of diagnostics involving up to 30 neuropathologists (Alafuzoff et al. 2008, 2009). Assessing neurodegenerative lesions, the regional distribution and the extent of IR are of major significance. It was observed that the agreement rates while assessing the same stained section were as high as 80–90% (Alafuzoff et al. 2009). If the stainings had been carried out in different laboratories, the agreement rates dropped, ranging 50–80% (Alafuzoff et al. 2008). If, however, also the ST would have varied, the agreement rates based on results here and BNEs result would probably have been extremely low.

As previously reported, the ST is not the only parameter to be considered when using IHC technique. When examining PM tissue, a variable such as the agonal state is described, influencing tissue characteristics mediated by pH changes (Monoranu et al. 2009). Following cold-ischemia time and PMD also alters the IHC outcome by induced autolysis and enzymatic activation (Espina et al. 2009; Dennighoff et al. 2017; Lundström et al. 2018).

The tissue is placed in various fixative solutions, and aldehyde-based fixatives are commonly used (Paavilainen et al. 2010; Engel and Moore 2011; Ramos-Vara and Miller 2014). The standard method is fixation with 10% neutral buffered formalin (4% formaldehyde) at room temperature. The chemical process of formalin fixation includes formation of cross-links between proteins, through covalent bonds. This alters the ability of Abs to bind to antigens by blocking their epitopes and changing their molecular structure (Leong et al. 2010; Ramos-Vara and Miller 2014). Additional factors to consider regarding fixative include the FT, temperature and pH of the fixative. Over and under fixation has been reported to alter IR while applying IHC (Boenisch 2005; Pikkarainen et al. 2010; Ramos-Vara and Miller 2014; Lundström et al. 2018).

The choice of embedding medium is also relevant, as melting temperature and duration of paraffin baths can alter the IHC outcome (Engel and Moore 2011).

There are several studies describing good preservation of antigens in tissue archived in paraffin blocks over decades, whereas storage of tissue sections has been reported to reduce the IR (Engel and Moore 2011; Karlsson and Karlsson 2011; Ramos-Vara and Miller 2014; Grillo et al. 2015, 2017).

There are several AR methods, including enzymatic and heat-induced. With these AR methods the protein cross-links formed by formalin fixation are degraded (Boenisch 2005; Leong et al. 2010; Ramos-Vara and Miller 2014). When needed, combinations of various AR techniques are implied (Alafuzoff et al. 2009).

The choice of Ab is crucial for the outcome of IHC. There are thousands of commercial Abs available on the market. Various Abs have to be tested and validated before use in diagnostic or research settings (Vyberg and Nielsen 2016). Majority of Abs are of IgG type. Monoclonal Abs display higher specificity, while polyclonal Abs exhibit higher affinity due to multiple epitopes. Polyclonal Abs are thus more prone to cross-reactions with other proteins, resulting in unspecific background staining (Leong et al. 2010; Ramos-Vara and Miller 2014). Noteworthy, the molecular mass of the IgG molecule ranges from 150 to 170 kDa for a species, and the dimension is 4 × 8.5 × 14.5 nm (Tan et al. 2008). Addition of a specific molecule while constructing an Ab will certainly increases the mass and dimension of the molecule. Information regarding the size of different commercial Abs is not available, but it is probable that the size of the Ab will influence the diffusion of the Ab into the section. In 2017 McCampbell and colleagues pointed out that the use of ST outside vendors recommendations might change the intensity, number of IR cells and eventually overall diagnosis. This significant relationship between an Ab and ST is not all acknowledged. Thus, not only the thickness of the section, but also the size of the Abs might influence the staining outcome.

DS are used to visualise the antigen binding by enzyme-chromogen mediated reaction. The technique has evolved from one-step, i.e. one Ab reacts with an antigen, to 2 or 3 steps. Currently, the polymer based DS, where several secondary Abs are bound to a polymer with multiple enzyme/chromogen molecules, is used. In summary, the choice of DS is also relevant for the outcome of IHC (Skaland et al. 2009; Ramos-Vara and Miller 2014). The size of molecules used in DS might also be influence the diffusion into the three-dimensional tissue and thus the outcome.

Standardised protocols while applying IHC are required to obtain consistent and reproducible results both within a laboratory, but also in inter-laboratory settings (Vyberg and Nielsen 2016). Today, automatic IHC platforms are often used to limit human error, thus, allowing for consistent staining results to be obtained (Ramos-Vara and Miller 2014). Noteworthy, however, regarding the automatic systems, there are significant limitations (factory programme settings, scanty information regarding solutions used, ready-to-use Abs) that are poorly acknowledged.

In research settings, the manual staining is often the mode of action, as the transparency of the methodology is fundamental when the goal is to replicate an experiment. Noteworthy, the IHC outcome varies significantly while implementing manual staining and/or various automatic systems (Alafuzoff et al. 2008).

In conclusion, there is a long list of factors influencing the IHC outcome. Here, we report that the ST should also be considered as well as the molecular size of the Ab. Recently, Nosek and Errington described that substantial number of reports in the scientific field cannot be replicated (2017). Regarding the field of pathology, the IHC method, based on the above, is probably the most unpredictable and an eventual source of error.
